# Acute multivessel coronary artery occlusion: a case report

**DOI:** 10.1186/1756-0500-5-523

**Published:** 2012-09-24

**Authors:** Feng Gan, Dongnan Hu, Tianran Dai

**Affiliations:** 1 Cardiology Care Unit, Beijing General Aerospace Hospital, Beijing, 100076, China

**Keywords:** Interventional cardiology, Myocardial infarction, Multivessel coronary artery

## Abstract

**Background:**

In terms of clinical and angiographic findings, multiple simultaneous coronary occlusions in acute myocardial infarction are infrequent, and the mechanism of the occlusions is unclear.

**Case presentation:**

We herein report a rare case of two simultaneously occluded coronary arteries, one of which subsequently underwent spontaneous lysis. An 88-year-old man had a 3-hour attack of acute crushing retrosternal chest pain. His first electrocardiogram showed ST-segment elevation in the inferior (II, III, and aVF) and anterior (V3–V6) leads. His second electrocardiogram in the cardiac care unit showed ST-segment elevation in the inferior leads but ST-segment depression in the anterior leads. Emergency coronary angiography revealed that the right coronary artery was acutely and totally occluded at the midportion and that the proximal and midportion of the left anterior descending coronary artery had an acute thrombus. According to his electrocardiogram and coronary angiography findings, we inferred that the right coronary artery and left anterior descending coronary artery first totally occluded simultaneously, and then the thrombus in the left anterior descending coronary artery spontaneously underwent partial lysis. Therefore, intervention of the right coronary artery was performed followed by injection of glycoprotein IIB-IIIA inhibitor into the left anterior descending coronary artery. He had an uneventful hospital course and was discharged home 10 days later.

**Conclusion:**

Because patients with multivessel coronary artery occlusion are often in serious condition, abnormal electrocardiographic results must be identified and affected vessel should be opened timely and efficiently to save the myocardium and reduce complications such as congestive heart failure.

## Background

In terms of clinical and angiographic findings, multiple simultaneous coronary occlusion in acute myocardial infarction is infrequent. Fewer than 30 cases have been reported [1]. The mechanism of multiple simultaneous coronary occlusions is not clear. In the literature, several factors are thought to be closely correlated with the disease, such as essential thrombocythemia, multivessel spasm, hypercoagulability, and cocaine abuse. Moreover, traditional coronary heart disease risk factors such as diabetes mellitus, cigarette use, and hyperlipidemia also contribute to occlusion. Whether multiple simultaneous coronary occlusion is one special type of coronary heart disease or a haphazard incident is unclear.

## Case presentation

An 88-year-old male patient arrived at our emergency department with a 3-hour history of acute crushing retrosternal chest pain. The chest pain began while he was asleep. He had no history of spontaneous bleeding, smoking, hyperlipidemia, diabetes mellitus, atrial fibrillation, or family history of coronary artery disease. However, he had hypertension and underwent tracheotomy because of laryngocarcinoma 7 years previously.

The physical examination showed that his respiratory rate was 22/min, heart rate was 46/min, and blood pressure was 110/70 mmHg. His neck veins were not distended, and his breath sounds were clear. His heart rhythm was regular and heart sounds were clear with no murmurs. Peripheral pulses were intact with no edema.

**Figure 1:**
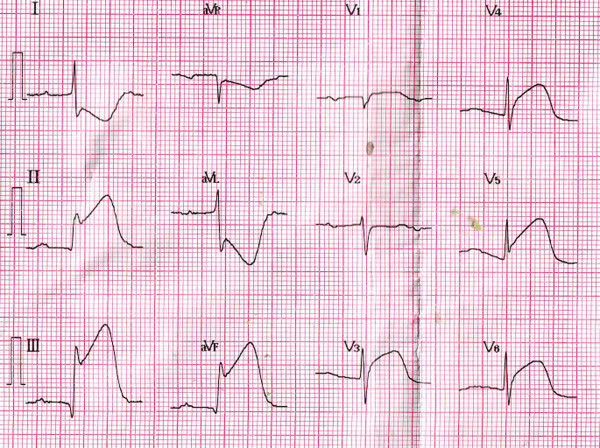
**Initial electrocardiogram.** Shown is ST-segment elevation in the inferior (II, III, and aVF) and anterior (V3–V6) leads.

**Figure 2:**
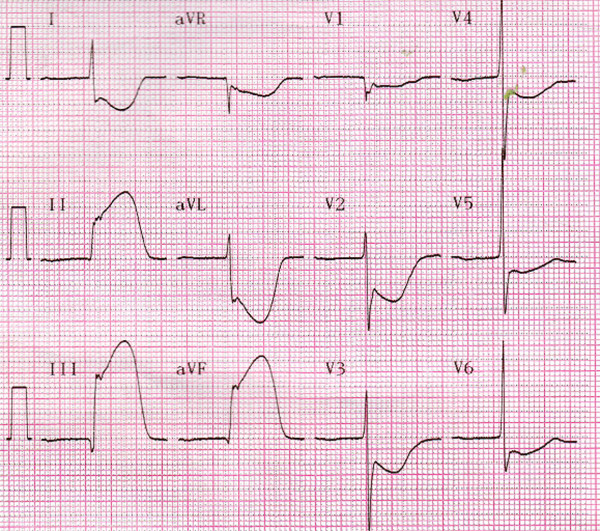
**Second electrocardiogram in the cardiac care unit.** Shown is ST-segment elevation in the inferior leads but ST-segment depression in the anterior leads.

His initial electrocardiogram (ECG) showed ST-segment elevation in the inferior (II, III, and aVF) and anterior (V3–V6) leads (Figure 1), which was considered to indicate acute inferior and anterior myocardial infarction. However, his second ECG in the cardiac care unit had changed to show ST-segment elevation in the inferior leads but ST-segment depression in the anterior leads (Figure 2).

**Figure 3:**
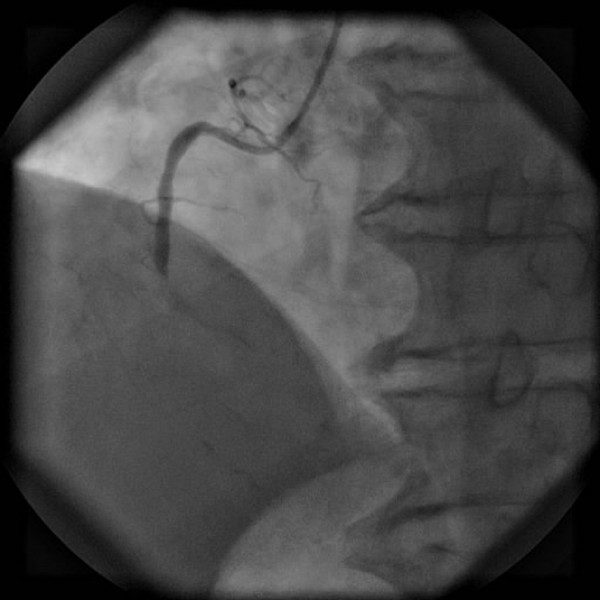
**Coronary angiography.** The right coronary artery was acutely and totally occluded at the midportion.

**Figure 4:**
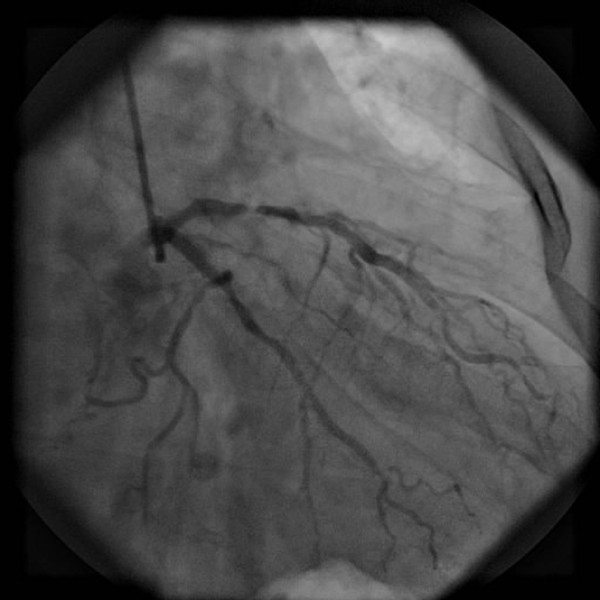
**Coronary angiography.** The proximal and midportion of the LAD coronary artery had a hazy filling defect, which suggested an acute thrombus.

**Figure 5:**
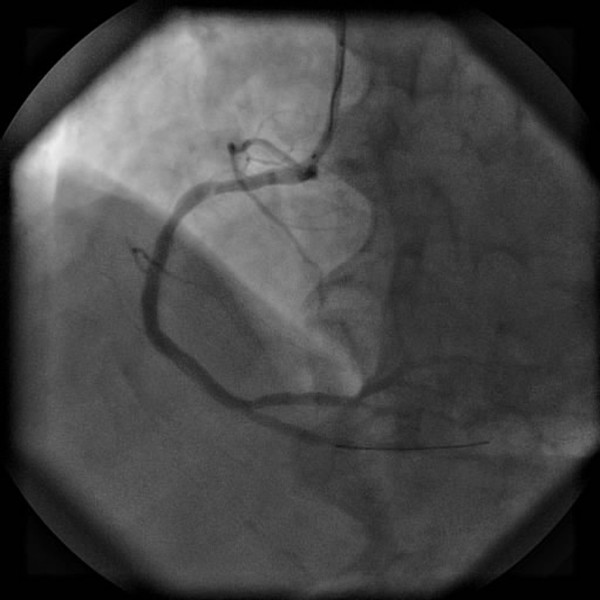
Image after intervention of the right coronary artery.

**Figure 6:**
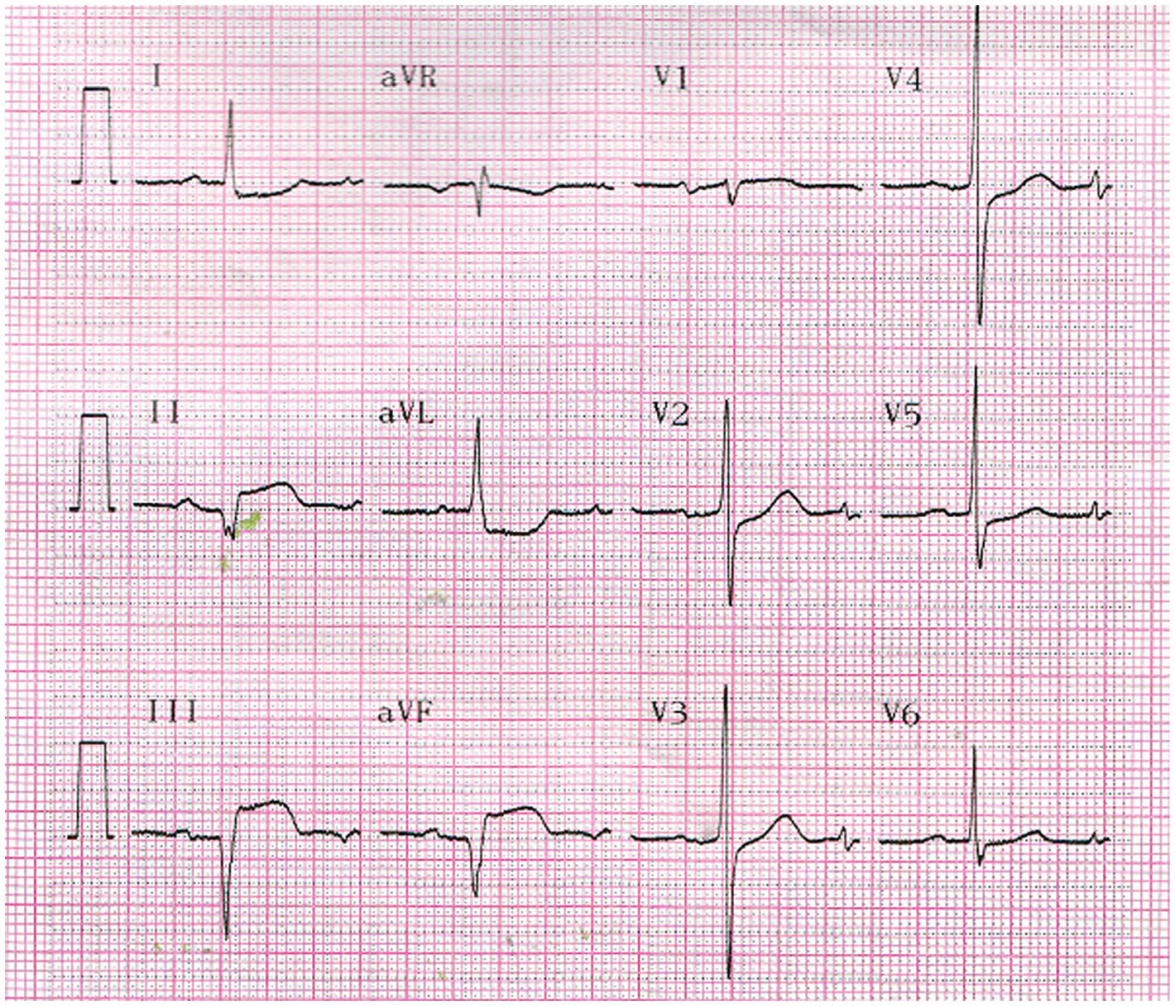
Electrocardiogram before discharge from the hospital.

The patient underwent primary angioplasty after receiving aspirin (100 mg) and clopidogrel (300 mg). Coronary angiography revealed that the right coronary artery (RCA) was acutely and totally occluded at the midportion (Figure 3) and that the proximal and midportion of the left anterior descending coronary artery (LAD) had a hazy filling defect, which suggested an acute thrombus (Figure 4). We inferred that the RCA and LAD had first totally occluded simultaneously, followed by spontaneous partial lysis of the LAD thrombus. Intervention of the RCA was performed first (Figure 5), and only because of TIMI III flow in the LAD was glycoprotein IIB-IIIA inhibitor subsequently injected into the LAD. The patient’s symptoms and postoperative ECG results were significantly improved (Figure 6). After the operation, the patient received antiplatelet and antithrombotic therapy, aspirin, clopidogrel, low-molecular-weight heparin, and glycoprotein IIB-IIIA inhibitor.

ECG after the procedure showed an ejection fraction of 54% with mild hypokinesia of the inferior walls. Creatine kinase-MB fraction levels peaked 12 hours later at 310 ng/mL (normal, 0–4.3 ng/mL). After the procedure, the patient had an uneventful course with no evidence of pulmonary congestion or recurrence of chest pain. The patient was discharged home 10 days later on aspirin, clopidogrel, a statin, a beta blocker, and an angiotensin converting enzyme inhibitor.

## Conclusions

Multiple simultaneous coronary occlusions in acute myocardial infarction are infrequent with fewer than 30 case reports published in the literature [1]. However, some reports contain no reliable ECG evidence. Acute and chronic occlusions are confused because they are based only on angiographic findings. In the present case, ECG and angiographic findings both supported multiple simultaneous coronary occlusions. The two ECGs had 20-minute intervals, but the results were strikingly different. The initial ECG showed ST-segment elevation in the inferior (II, III, and aVF) and anterior (V3–V6) leads (Figure 1); however, the second ECG showed ST-segment elevation in the inferior but ST-segment depression in the inferior leads (Figure 2). Coronary angiography revealed that the RCA was acutely and totally occluded at the midportion (Figure 3) and that the proximal and midportion of the LAD had a hazy flocculent shadow that suggested an acute thrombus (Figure 4). We believe that acute total simultaneous occlusions occurred at the RCA and LAD before presentation to the hospital, and then the thrombus in the LAD had lysed on the way to the care unit. We believe that this is the best interpretation of the patient’s ECG changes.

Multiple simultaneous coronary occlusions in acute myocardial infarction always lead to very serious illness. More than 50% of patients in previous reports presented with cardiogenic shock [1]. Therefore, early diagnosis and proper treatment are the most important factors in the therapeutic process.

Early diagnosis involves the timely and accurate performance of ECG. At the same time, skillful analysis of coronary angiography images should be carried out. According to the previous literature [2], acute thrombosis in the coronary arteries have the following features: (1) a hazy, transformable, or flocculent appearance at the site of occlusion; (2) intimal discontinuity or anomalism; and (3) globular filling defects. Furthermore, passing of the occlusion can be attempted with a soft wire if necessary. Acute thrombi are always softer than chronic thrombi.

Because no instances of myocardial infarction due to multiple simultaneous occlusions have been described in the published series [3-5], no accordant advice can be given in the treatment of these cases. Thrombolysis, stents, angioplasty, medical management, thrombectomy, and aspiration thrombectomy have been proven effective in previous cases. According to published guidelines, careful analysis of every patient’s condition is mandatory. In the present patient, intervention of the RCA was performed first, and only because the LAD flowed in TIMI III. With respect to the LAD thrombus burden, glycoprotein IIB-IIIA inhibitor was injected into the LAD and then into the vein. This treatment is valid.

In summary, multiple simultaneous coronary occlusions in acute myocardial infarction is infrequent. Multiple mechanisms may lead to this situation, including cocaine abuse, essential thrombocythemia, and others. This patient had no risk factors for coronary artery disease, and his condition may be attributed to random rupture of multiple plaques. Patients with multivessel occlusive coronary artery disease are often in a serious condition. Careful attention should be given to identification of abnormal ECG results. Accurate identification of the affected vessels should be achieved with ECG and coronary angiography results without misdiagnosis or missed diagnosis. The affected vessel should be opened timely and efficiently in an effort to save the myocardium and reduce complications such as congestive heart failure.

## Consent

Written informed consent was obtained from the patient for publication of this Case Report and any accompanying images. A copy of the written consent is available for review by the Editor-in-Chief of this journal.

## Competing interests

The authors declare that they have no competing interests.

## Authors’ contributions

FG carried out the treatment of the patient and drafted the manuscript. DH performed the percutaneous coronary intervention. TD participated in the treatment and helped to draft the manuscript. All authors read and approved the final manuscript.

## References

[B1] Kanei Y Janardhanan R Fox JT Gowda RM Multivessel Coronary Artery Thrombosis: Literature Review J Invasive Cardiol 2009 21 3 66 68 19182293

[B2] Shen AY-J Mansukhani PW Aharonian VJ Jorgensen MB Primary Angioplasty for Acute Myocardial Infarction Resulting From the Simultaneous Occlusion of Two Major Coronary Arteries Catheter Cardiovasc Interv 1999 47 203 207 10.1002/(SICI)1522-726X(199906)47:2<203::AID-CCD17>3.0.CO;2-X 10376506

[B3] Grines CL Browne KF Marco J Rothbaum D Stone GW O’Keefe J Overlie P Donohue B Chelliah N Timmis GC Vlietstra RE Strzelecki M Puchrowicz-Ochocki S O’Neill WW Acomparison of immediate angioplasty with thrombolytic therapy for acute myocardial infarction N Engl J Med 1993 328 673 679 10.1056/NEJM199303113281001 8433725

[B4] O’Keefe JO Bailey WL Rutherford BD Hartzler GO Primary angioplasty for acute myocardial infarction in 1,000 consecutive patients Am J Cardiol 1993 72 107G 115G 10.1016/0002-9149(93)90115-S 8279345

[B5] Ellis SG O’Neill WW Bates ER Walton JA Jr Nabel EG Werns SW Topol EJ Implications for patient triage from survival and left ventricular functional recovery analyses in 500 patients treated with angioplasty for acute myocardial infarction J Am Coll Cardiol 1989 13 1251 1259 10.1016/0735-1097(89)90296-9 2522954

